# IBD and Immune-Mediated Inflammatory Diseases: What Is the Optimal Management?

**DOI:** 10.3390/jcm15124408

**Published:** 2026-06-06

**Authors:** Mohammad Alsaeid, Osamah Abu Hawi, Talat Bessissow, Peter L. Lakatos

**Affiliations:** 1 Division of Gastroenterology, McGill University, Montreal, QC H4A 3J1, Canada; mohammadalsaeid@gmail.com (M.A.); abuhawi2010@gmail.com (O.A.H.); talat.bessissow@mcgill.ca (T.B.); 2 Department of Internal Medicine and Oncology, Semmelweis University, 1083 Budapest, Hungary

**Keywords:** IBD, CD, UC, IMID, extraintestinal manifestations, dermatological manifestations, ophthalmologic manifestations, musculoskeletal manifestations, neurological manifestations, multidisciplinary approach

## Abstract

**Background/Objectives:** Inflammatory bowel diseases (IBD) including ulcerative colitis and Crohn’s disease can be associated with other immune-mediated inflammatory diseases (IMIDs) and extraintestinal manifestations (EIM) including dermatological manifestations, ophthalmologic manifestations, musculoskeletal manifestations and neurological manifestations. The aim of this narrative review is to discuss the optimal management and treatment strategy of IBD with immune-mediated inflammatory disease and extraintestinal manifestations. **Methods:** This review is based on published studies searched in PubMed until 31 December 2025. Our search focused on systemic reviews, review articles, randomized trials, cohort studies, guidelines and case series. **Results:** In IBD, the presence of additional immune-mediated inflammatory diseases (IMIDs) should be considered a poor prognostic factor, prompting closer disease monitoring and earlier escalation to or optimization of advanced therapy. Therapeutic management requires careful consideration including a multidisciplinary approach and a selection of the most appropriate treatment option(s) based on the presence, severity of IBD and/or IMIDs and/or EIM. For patients with refractory disease affecting multiple organs, emerging strategies include dual biologic therapies. **Conclusions:** The optimal management of IBD with associated IMIDs/EIM should be multidisciplinary in close cooperation among specialists to align treatment goals and management plans.

## 1. Introduction

Immune-mediated inflammatory diseases (IMIDs) are a heterogeneous group of autoimmune disorders with multifactorial etiologies, and patients with inflammatory bowel disease (IBD)—especially those with Crohn’s disease—have a higher prevalence of coexisting IMIDs such spondyloarthritis, psoriasis and uveitis [1,2,3]. A cohort study showed that the prevalence of IMIDs in patients with IBD is 26.6% [3]. Skin psoriasis and spondyloarthritis are some of the most common IMIDs associated with IBD [3]. The medical literature demonstrates that the presence of IMIDs in IBD patients is associated with a more aggressive course including higher rate of surgery, increased need for biological therapy and poorer quality of life [4]. Women are more likely to have IMIDs and risk is more pronounced in Crohn’s disease. These findings highlight that the IBD associated with IMIDs warrants closer monitoring and aggressive management [5]. It has been suggested previously that the management of IBD with associated IMIDs should be multidisciplinary with close coordination among specialists to align treatment plans [5]. In this narrative review we will focus on summarizing and giving guidance on treatment strategies and optimal management of IBD in the presence of different IMIDs and challenging EIMs.

## 2. Materials and Methods

This review is based on pertinent publications retrieved by literature search in PubMed. The search terms include “Inflammatory bowel disease”, “IBD”, “Ulcerative colitis”, “UC”, “Crohn’s disease”, “CD”, “Treatment”, “Management”, “Psoriasis”, “psoriatic arthritis”, “Spondyloarthritis”, “ankylosing spondylitis”, “Multiple Sclerosis”, “Hidradenitis suppurative”, “Uveitis”, “Erythema nodosum”, “Pyoderma gangrenosum”, “Rheumatoid arthritis”, “Episcleritis”, “Scleritis”, “primary sclerosing cholangitis” and “PSC”. Our search focused on systemic reviews, review articles, randomized trials, cohort studies, guidelines and case series until 31 December 2025. The authors of this review evaluated and summarized these publications and contributed as well from their clinical knowledge.

## 3. Efficacy Signal for Medications in IBD and IMIDs/EIMs

The optimal management involves multidisciplinary collaborative care teams including a dermatologist, rheumatologist, ophthalmologist and gastroenterologist (IBD expert) to help ensure optimal treatment strategy is implemented based on the leading clinical symptom, and the severity of the IBD and/or IMIDs [6].

Differentiating between true extraintestinal manifestations (EIMs) of IBD and co-existing immune-mediated inflammatory diseases (IMIDs) is a well-recognized clinical challenge and the distinction between both is blurred for several reasons including overlapping pathophysiology, genetic susceptibility and clinical presentations [7]. IBD patients have higher rates of psoriasis and MS compared to the general population which suggests that EIM/IMIDs are not coincidental [8].

Table 1 below summarizes the organ system-based classification of both classic EIMs and associated IMIDs in IBD.

Table 2 below summarizes the medical therapies approved for treatment of IBD, and the evidence available on its efficacy in the possible coexisting IMIDs (psoriasis, ankylosing spondylitis, psoriatic arthritis, MS, uveitis, rheumatoid arthritis), as well as the proposed mechanism of action of each treatment, while the safety profile of advanced therapies is summarized in Table 3.

Furthermore, findings from the VEGA study highlight that combination of novel biological therapy (guselkumab and golimumab) can achieve superior therapeutic efficacy both endoscopically and clinically compared to monotherapy in biologically naive moderate-severe ulcerative colitis. This study suggests that advanced combination therapy could be a potential future treatment strategy [77] and it may be associated with superior control for IBD-associated IMIDs/EIMs as well.

## 4. Management and Treatment Strategy in Patients with IBD and IMIDs/EIMS

Various immune-mediated inflammatory diseases (IMIDs) occur with increased frequency in IBD, including psoriatic arthritis, ankylosing spondylitis and psoriasis [78,79]. These associations are thought to reflect shared immunogenetic pathways, including HLA class I-related mechanisms and ERAP-1-dependent antigen processing abnormalities [80,81]. Current evidence does not consistently demonstrate an association for these IMIDs with a specific disease phenotype (e.g., according to the Montreal classification) [78,82].

In contrast, based on the evidence from published population-based cohorts, extraintestinal manifestations (EIMs), like type 1 peripheral arthritis, may be associated with specific IBD phenotypes. For example, higher prevalence of type 1 peripheral arthritis in colonic Crohn’s disease and in stricturing/penetrating disease [9]. Similarly, the rate of EIMs may increase with an increase in the extent of UC disease and IBD patients with already one EIM may be at risk to present with additional EIMs [9].

Furthermore, in ulcerative colitis (UC) the presence of extraintestinal manifestations is associated with a more severe disease course including extensive disease, higher steroid requirement and higher hospitalization. However, Crohn’s disease with EIMs is associated with higher treatment intensity (increased steroid use or azathioprine use) but not associated with increased surgical rate or hospitalization [83].

The optimal management involves multidisciplinary collaborative care teams including a dermatologist, rheumatologist, ophthalmologist and gastroenterologist (IBD expert/hepatologist) to help ensure optimal treatment strategy is implemented based on the leading clinical symptom, and the severity of the IBD and/or IMIDs [6].

Finally, PSC is thought to be more prevalent in UC and associated with a unique ‘PSC–IBD’ phenotype [84,85]. The PSC–IBD phenotype is characterized by right-sided predominance, relative rectal sparing, increased backwash ileitis, and elevated colorectal neoplasia risk [86,87,88]. Of note, in this review authors focus on the management of coexisting IMIDs/ EIMs in IBD patients.

### 4.1. IMIDs and IBD

#### 4.1.1. Psoriasis (Ps/PsA) and IBD

The incidence of inflammatory bowel disease in psoriasis is increased with a higher incidence of Crohn’s disease rather than ulcerative colitis. The prevalence of psoriasis in Crohn’s disease patients and ulcerative colitis patients in published cohorts ranges from 3.6 to 6.6% and 2.8–4.8%, respectively [89,90,91]. In comparison, the prevalence of psoriasis in the general population is 2.3% [89,90,91]. The incidence of psoriasis in ulcerative colitis patients and Crohn’s disease patients ranges from 0.218% and 0.229%, respectively [89,90,91].

Psoriasis and Crohn’s disease have a strong genetic predisposition and genome-wide association studies were able to identify 11 genes of predisposition common in both psoriasis and IBD [92].

As treatment options, anti-TNF agents (specifically infliximab, adalimumab, golimumab and certolizumab pegol) are recommended first-line biologics as they are effective for both psoriasis and IBD, see advanced treatment options in Table 4 [92]. The EXPRESS trial showed that infliximab is one of the most effective anti-TNF agents for psoriasis [21]. A total of 80% of psoriasis patients achieved PASI 75 with infliximab at week 10 in comparison to 2% of psoriasis patients achieving PASI 75 with placebo at week 10 [21].

Similarly, anti-IL12/23 (ustekinumab) and IL-23 inhibitors (risankizumab, guselkumab) are both effective for treating IBD with coexisting psoriasis. However, risankizumab has demonstrated superior efficacy to ustekinumab in treating psoriasis [93]. The UltIMMa-1 and UltIMMa-2 trials showed that 75.3% of moderate-severe psoriasis patients who received risankizumab reached PASI 90 at week 16 in comparison to 42% of moderate-severe psoriasis patients who received ustekinumab reached PASI 90 at week 16 and 4.9% patients who received placebo [48].

IL-23 inhibitors demonstrate the most favorable safety profile, while anti-TNF agents have the highest risks of serious infections and ustekinumab shows an intermediate risk profile with no increased risk of malignancy or serious infections [90].

The first-line advanced therapy for managing psoriatic arthritis with concomitant active IBD is anti-TNF agents based on ACR 2018 guidelines [94]. The IMPACT 2 trial showed the effectiveness of anti-TNF agents (infliximab) for psoriatic arthritis. A total of 58% of psoriatic arthritis patients who received infliximab achieved ACR 20 at week 14 in comparison to 11% in the placebo group, and 64% of patients receiving infliximab achieved PASI 75 at week 14 in comparison to 2% in the placebo group [95].

Other advanced therapies in the management of psoriatic arthritis with concomitant IBD include IL23p19 inhibitor (risankizumab and guselkumab) and JAK inhibitors (Upadacitinib). The Discover-1 trial showed the effectiveness of IL23p19 (guselkumab) for psoriatic arthritis. In total, 59% of psoriatic arthritis patients who received guselkumab achieved ACR 20 at week 22 in comparison to 22% in the placebo group (*p* < 0.001) [96]. The SELECT PsA-1 trial showed upadacitinib 30 mg is superior to adalimumab in managing psoriatic arthritis. In total, 78% of psoriatic arthritis patients who received upadacitinib 30 mg achieved ACR 20 at week 12 in comparison to 36% of placebo group and 65% of adalimumab group (*p* < 0.001) [65]. The ACT1 trial demonstrated the common adverse events in response to anti-TNF (maintenance infliximab) which included upper respiratory tract infection (23.8%), arthralgia (17.2%), headache (14.8%), sinusitis (13.1%), and pharyngitis (11.5%) [72]. The VOYAGE 1 and VOYAGE 2 clinical trials showed that the adverse events related to IL23p19 inhibitors (risankizumab) include nasopharyngitis (23.4%), upper respiratory tract infection (15.4%), headache (6.8%), arthralgia (9%), and back pain (6%) [73]. 

Anti-IL-17 agents are among the most effective advanced therapy for psoriasis. However, anti-IL-17 agents are contraindicated in patients with co-existing IBD due to the risk of IBD exacerbation [29,97].

#### 4.1.2. Rheumatoid Arthritis and IBD

Rheumatoid arthritis occurs more frequently in patients with inflammatory bowel disease than in the general population. A meta-analysis showed a relative risk of rheumatoid arthritis in IBD patients of approximately 2.59 [98], though specific prevalence and incidence percentages are not well-identified in the literature. 

Treatment options of rheumatoid arthritis with IBD include steroids, sulfasalazine, methotrexate, azathioprine and advanced biological therapy. The advanced biological therapy options for rheumatoid arthritis with IBD include anti-TNF agents and JAK inhibitors, see also in Table 5. 

The ATTRACT trial showed that 50–58% of rheumatoid arthritis patients who received both infliximab and methotrexate achieved ACR20 in comparison to 20% of the placebo-methotrexate group [99]. The SELECT-COMPARE trial showed that 71% of rheumatoid arthritis patients who received upadacitinib reached ACR20 at week 12 compared to 36% in the placebo group [100]. 

Avoided treatments: IL-17 inhibitors should be avoided in rheumatoid arthritis with coexisting IBD as it might cause exacerbation of intestinal inflammation [101].

Anti-IL-17 agents (secukinumab, ixekizumab, bimekizumab) are generally suggested to be avoided in IBD as it can induce IBD or worsen a co-existing IBD [39].

Etanercept is also an TNF alpha inhibitor. However, it is not effective in IBD and therefore should be avoided if rheumatoid arthritis coexists with IBD [101]. Moreover, some data suggest that its use may be associated with and increased risk for developing IBD [102]. In a population-based study from Denmark by Korzenik et al. etanercept, but not infliximab or adalimumab, use was associated with an app 2-fold elevation of developing both CD and UC in patients treated for autoimmune disease over a period of 14 years [102].

#### 4.1.3. Multiple Sclerosis and IBD

There is an increased risk of comorbidity between multiple sclerosis (MS) and inflammatory bowel disease (IBD) with meta-analyses showing 50% increased risks of developing IBD or MS in the presence of the other regardless of the type of IBD (Crohn’s disease or Ulcerative colitis) [103]. The prevalence of IBD in multiple sclerosis (MS) patients is 0.1–1.6% before diagnosis of MS and 0.36–4.6% after diagnosis of MS [103]. The course of MS is milder in patients with both MS and IBD in comparison to isolated MS [104].

Possible treatment options are natalizumab and ozanimod. natalizumab (alpha-4 integrin antagonist) is approved for both moderate-severe Crohn’s disease and relapsing MS, see also in Table 6. However, it requires careful monitoring for progressive multifocal leukoencephalopathy and it is generally not used in IBD [23].

The AFFIRM trial for multiple sclerosis showed that relapsing-remitting multiple sclerosis patients who were on natalizumab after one year had reduced the annualized rate of relapse to 0.26 relapse per year in comparison to 0.81 relapse per year in the placebo group (*p* < 0.001) [105].

Sphingosine-1-phosphate (S1P) modulators such as ozanimod are also approved for both relapsing MS and active moderate-severe ulcerative colitis [23].

In the SUNBEAM clinical trial, relapsing multiple sclerosis patients who received ozanimod 1 mg had a lower annualized relapse rate of 0.18 in comparison to interferon beta 1a group 0.35 (*p* Value < 0.0001) [106]. 

The SUNBEAM clinical trial showed the adverse events of ozanimod included any infection (10.7%), nasopharyngitis (3.5%), headache (3.3%), increased transaminases (2.6%), and arthralgia (2.3%) [106].

The AFFIRM trial showed the adverse events of natalizumab include headache (38%), fatigue (27%), urinary tract infection (20%), arthralgia (19%), depression (19%), lower respiratory tract infection (19%) and rash (11%) [105]. 

A retrospective analysis of data from four clinical studies showed the estimated risk of progressive multifocal leukoencephalopathy (PML) in MS patients receiving natalizumab can be stratified based on three risk factors: previous immunosuppressant use, anti-JCV antibodies in serum, and treatment duration. The estimated risk of PML in anti-JCV antibodies negative was less than 0.07 per 1000 patients. In anti-JCV antibodies positive patients with previous immunosuppression, the estimated PML probability over 6 years was 2.7%. In anti-JCV antibodies positive patients with no previous immunosuppression, the estimated PML probability over 6 years was 1.7% [107]. 

Finally, Azathioprine can be used for mild MS and mild IBD [23].

Of note, anti-TNF agents are contraindicated in MS and should be avoided as there is associated risk of possible demyelination and worsening of MS [23].

#### 4.1.4. Hidradenitis Suppurative (HS) and IBD

Hidradenitis suppurative (HS) and inflammatory bowel disease are bidirectionally associated chronic inflammatory conditions that share common pathogenic mechanisms and therapeutic approaches. The prevalence of HS among IBD patients ranges up to 1.3–2% with higher rates in Crohn’s disease in comparison to Ulcerative colitis [108].

HS is characterized by recurrent abscess, painful nodules, draining sinuses and scarring in intertriginous areas [108]. The diagnosis/differential diagnosis requires disease anatomic distribution along with lesion morphology (nodules, abscesses and scars) and disease chronicity (two recurrences within 6 months or persistent lesions more than 3 months) [109]. The most common anatomic distribution sites for HS include axilla, groin, anogenital region and inframammary folds [109].

Patients with concomitant HS and IBD have a more severe disease phenotype.

Treatment approaches for HS vary based on severity of disease, see in Table 7. Mild HS is treated with topical clindamycin [109]. Moderate disease requires oral tetracycline and topical clindamycin [109]. Adalimumab is the biological therapy of choice for treating moderate-severe disease [109]. The PIONEER 1 trial showed that 41.8% of patients with HS who received adalimumab weekly reached HS clinical response (HiSCR) by week 12 in comparison to 26% of patients who were on placebo (*p* = 0.003) [110]. Infliximab is a reasonable second-line alternative option when adalimumab fails. One small randomized clinical trial showed patients who received infliximab (27%) in comparison to patients who received placebo (5%) achieved 50% or greater of HS severity Index score reduction at 8 weeks [111]. Ustekinumab showed promising but statistically nonsignificant improvement [112]. Surgical options include punch debridement and unroofing for localized disease. Wide excision is reserved for extensive and severe disease with scarring [109].

### 4.2. EIM and IBD

#### 4.2.1. Spondyloarthritis (SpA)/AS and IBD

Spondyloarthritis (SpA) consists of ankylosing spondylitis, psoriatic arthritis, reactive arthritis and inflammatory bowel disease-associated spondyloarthritis (IBD-SpA) [113]. SpA is regarded as EIM, yet is estimated to occur in 10–20% of IBD patients [113]. The prevalence of ankylosing spondylitis in IBD patients is approximately 3% [114]. IBD and SpA share a common signaling pathway with some studies showing that the IL23 IL17 axis is involved in the pathogenesis of both IBD and SpA [113]. 

Conventional DMARDs (sulfasalazine and methotrexate) can be used to treat both peripheral SpA and IBD. However, sulfasalazine and methotrexate are not proven to be effective for treating axial SpA [115]. NSAID is the first-line therapy for axial SpA; however, NSAIDs are suggested to be avoided since they can cause IBD flare [115].

Anti-TNF agents are recommended over other biological therapy for treating both IBD and SpA (peripheral and axial), see details in Table 8 [101]. It has been shown that four anti-TNF agents (adalimumab, certolizumab, golimumab and infliximab) are effective in treating both inflammatory bowel disease and ankylosing spondylitis. The ASSERT trial showed that 60% of patients who received infliximab achieved ASAS20 responses at week 24 compared to 18% in the placebo group [116]. Etanercept was not effective in the treatment of IBD [39].

JAK inhibitors (tofacitinib and upadacitinib) have also been shown to be effective for both ankylosing spondylitis and IBD [39]. The SELECT-AXIS 1 trial showed that 52% of patients who received upadacitinib achieved ASAS40 responses at week 14 compared to 26% with placebo [66]. Deodhar et al. conducted a trial that showed 56.4% of ankylosing spondylitis patients on tofacitinib 5 mg reached ASAS20 at week 16 in comparison to 29.4% in the placebo group (*p* < 0.0001) [60]. In the OCTAVE sustain trial, ulcerative colitis remission at 52 weeks occurred in 40.6% of the patients in the 10 mg tofacitinib group, 34.3% in the 5 mg tofacitinib group versus 11.1% in the placebo group [57]. The U-ACCOMPLISH and U-ACHIEVE clinical trials showed that the common adverse events related to upadacitinib induction (45 mg) include nasopharyngitis (5%), CPK elevation (5%), acne (5%), upper respiratory tract infection (3%), arthralgia (2%) [48]. The common adverse events related to upadacitinib maintenance (15 mg) include nasopharyngitis (12%), CPK elevation (6%), acne (3%), upper respiratory tract infection (5%), arthralgia (6%) [62]. Avoided treatment: Anti-IL-17 agents (secukinumab, ixekizumab, bimekizumab) are effective for ankylosing spondylitis; however, it is generally suggested to be avoided in IBD as it can induce IBD or worsen a co-existing IBD [39].

#### 4.2.2. Erythema Nodosum and IBD

Erythema nodosum is characterized by tender, red, raised nodules most often in the anterior tibial area. However, it can be localized in the thighs and forearms. The prevalence of erythema nodosum in Crohn’s disease ranges 5–15% and 2–10% in ulcerative colitis patients [117]. 

In most cases, erythema nodosum is self-limiting and improves with IBD treatment with no subsequent scarring. If treatment is to be started, the aim should be to control the intestinal disease activity. Supportive treatment includes leg elevation, analgesic and compression stocking.

First-line treatment is corticosteroids and usually improvement with corticosteroids is fast. Anti-TNF is a rescue therapy, see advanced treatment options in Table 9 [118]. In a pooled analysis of 11 adalimumab clinical studies EIM resolution was noted in 54% adalimumab-treated patients compared to 31% in placebo group at 6 months, with a median time to initial resolution of cutaneous EIMs that was significantly shorter in the adalimumab group in comparison to the placebo group [119]. No clinical trial is available on the effectiveness of anti-TNFs for treating erythema nodosum, specifically. However, the systemic review by Peyrin-Biroute et al. pooled from nine interventional studies and 13 non-interventional studies showed that TNF antagonists achieved complete response for cutaneous manifestations including erythema nodosum with response rate 92–100% in the non-interventional studies (cohorts). The interventional studies showed TNF antagonists achieved complete response for cutaneous manifestation in pyoderma gangrenosum but not erythema nodosum with a response rate 21–25% [26].

Ustekinumab might also be effective in erythema nodosum associated with Crohn’s disease [120]. The post hoc analysis of the UNITI /IM-UNITI trials showed that erythema nodosum was the only EIM that was more likely to improve at week 52 with ustekinumab vs. placebo [121].

Treatment of erythema nodosum that is avoided in the context of IBD is long-term or frequent use of high-dose NSAID as it can cause IBD exacerbation [122]. 

UNIFI trial demonstrated the common adverse events of maintenance ustekinumab which include nasopharyngitis (16%), headache (4%), arthralgia (4.5%), and anemia (5.2%) [43]. 

#### 4.2.3. Pyoderma Gangrenosum and IBD

Pyoderma gangrenosum is characterized by rapidly evolving painful ulcerations and peripheral erythema. The prevalence of pyoderma gangrenosum in Crohn’s disease patients and ulcerative colitis patients are 2% and 1%, respectively [123,124]. The clinical course of pyoderma gangrenosum is unpredictable and may or may not correlate with IBD activity and may even present prior to IBD diagnosis [125]. However, pyoderma gangrenosum presents more often during active IBD [126]. 

Systemic corticosteroids have a rapid onset of action making it an excellent first-line option that results in resolution of 47% of cases of pyoderma gangrenosum by 6 months. 

Cyclosporin is a calcineurin inhibitor that results in resolution of 47% of cases of pyoderma gangrenosum in 6 months. Tacrolimus is a calcineurin inhibitor that is more potent than cyclosporin in treating pyoderma gangrenosum [127].

Infliximab is effective in treating Pyoderma gangrenosum with coexisting IBD, see also in Table 10. The Brooklyn et al. trial showed superiority of anti-TNF agents over placebo for the management of pyoderma gangrenosum. In total, 46% of pyoderma gangrenosum patients responded to infliximab in comparison to 6% in the placebo group at week 2 (*p* = 0.025), with 21% of patients reaching complete remission on infliximab at week 6 [25].

#### 4.2.4. Episcleritis/Scleritis and IBD

The most common ocular manifestation in IBD is episcleritis. Episcleritis seems to be more associated with active IBD than the other ocular manifestations [128]. Prevalence of episcleritis is up to 29% [123].

Sufficient treatment of intestinal inflammation is a key to treating episcleritis. Standard IBD treatments are used to control IBD with coexisting episcleritis. Topical corticosteroids lead to rapid improvement in episcleritis but it can be associated with adverse events including cataract formation and increased intraocular pressure [114]. Topical NSAID is ineffective [114]. 

Scleritis occurs in up to 18% of patients of Crohn’s disease and less frequently in ulcerative colitis [123]. Scleritis in IBD can precede, coincide with or follow intestinal symptoms [129]. 

Oral NSAIDs are the initial treatment for non-necrotizing anterior scleritis (diffuse and nodular) with an approximate of 7% of non-necrotizing scleritis patients failing to respond to initial oral NSAIDs [130]. The second-line option after failure to respond to oral NSAIDs is systemic oral steroids with an approximate of 16% of diffuse scleritis that fails to respond to oral steroids [130]. The third-line management option for scleritis is conventional immunomodulatory therapy (methotrexate and azathioprine) [130]. The fourth-line option is anti-TNF agents. No randomized clinical trials available show the effectiveness of anti-TNF in scleritis. However, in a prospective study, Sharma et al. reported that 91% of patients with scleritis achieved remission while receiving TNF inhibitor therapy after a median treatment duration of 1.2 years [131].

Mild cases may respond to topical steroids only. However, moderate-severe scleritis or refractory scleritis will require systemic therapy. Anti-TNF agents are effective for both ocular and intestinal manifestation and are preferred for persistent scleritis or refractory scleritis, see advanced treatment options in Table 11. 

NSAID is usually avoided as a treatment of scleritis in the context of coexisting IBD as it might exacerbate IBD flare. 

#### 4.2.5. Uveitis and IBD

Ocular complications of IBD occur in up to 10% of cases. Although they are EIMs, they may represent a significant treatment challenge. Uveitis is one of the most common ophthalmological manifestations in IBD patients. The overall prevalence of uveitis in IBD patients is 2.4% [132]. The prevalence of uveitis in Crohn’s disease patients (3.27%) is higher than the prevalence of uveitis in ulcerative colitis patients (1.6%) [132]. The presence of uveitis is closely related to HLA-B27. Females are at higher risk of developing uveitis [133].

Diagnosis requires confirmation by an ophthalmologist typically with a slit lamp examination to differentiate uveitis from other inflammatory ocular conditions [37].

The initial treatment includes topical steroids which can be escalated to systemic steroids. In severe cases refractory to corticosteroids or contraindication to corticosteroids, antimetabolites such as methotrexate and azathioprine can be used [37].

Biological therapy is indicated in patients with moderate-severe uveitis or refractory uveitis despite antimetabolites and steroids with active IBD, see advanced treatment options in Table 12. Anti-TNF (adalimumab, infliximab, golimumab and certolizumab) are the preferred biological therapy and adalimumab has the strongest evidence of effectiveness [37,134]. The VISUAL 1 trial showed a 50% reduction in the risk of treatment failure compared to placebo in patients with active non-infectious intermediate, posterior or panuveitis (*p* < 0.001); the primary efficacy result showed median time to treatment failure was 24 weeks with adalimumab vs. 13 weeks with placebo [135]. 

#### 4.2.6. Primary Sclerosing Cholangitis and IBD

IBD is highly prevalent in PSC patients and it is estimated that 70–80% of PSC patients have concomitant IBD or will develop IBD in the future [136]. The pooled prevalence of PSC in IBD is approximately 2.6% [137]. The typical clinical course of IBD in PSC patients is usually mild and responds well with mesalamine. However, less commonly the IBD is moderate to severe and requires the start of advanced therapy [136]. 

Management of IBD with coexisting PSC will follow the IBD management guidelines. There is no specific medical treatment that has been proven to alter the natural history of PSC or prolong transplant-free survival. Moderate doses of Ursodeoxycholic acid have been extensively studied; it may show improvement in liver blood biochemistry; however, the long-term benefit of Ursodeoxycholic acid is unclear. Immunosuppressive therapy has not been shown to improve outcome in classic PSC [138]. Annual colonoscopy with targeted biopsies targeted with chromoendoscopy is recommended for all PSC-IBD patients including post-liver transplant given the increased risk of colorectal cancer in PSC-IBD patients. Annual gallbladder ultrasound is advised and cholecystectomy should be performed if there is gallbladder mass [139]. 

High doses of Ursodeoxycholic acid should be avoided as there may be an increased risk of adverse outcome of death, progression to cirrhosis and cholangiocarcinoma associated with the high dose of ursodeoxycholic acid [140,141].

## 5. Expert Opinion

IBD patients with coexisting IMIDs may demonstrate more aggressive disease phenotypes with possibly more aggressive disease courses that require integrated multidisciplinary management strategies and mandatory collaborative strategy with an IBD specialist, rheumatologist, dermatologist and ophthalmologist. The presence of IMIDs should be considered a poor prognostic factor necessitating closer monitoring and early escalation to or optimization of advanced therapy.

The primary treatment choice should be dictated by the relative severity of the IBD vs. IMID in a multidisciplinary approach, where the lead is taken by the discipline with the more severe disease phenotype/course. 

Therapeutic management requires careful consideration of treatment options. Anti-TNF agents (adalimumab, infliximab, golimumab, certolizumab) remain the cornerstone of the therapy in most cases as they effectively treat both intestinal inflammation and as well in most IMIDs/EIMs. However, JAK inhibitors (tofacitinib and upadacitinib) and IL23 inhibitors (risankizumab and guselkumab) show a promising alternative choice or may be used as a second line after the failure to an anti-TNF-based approach in certain rheumatologic as well as skin conditions.

Furthermore, there are two main clinical scenarios for the rationale of using advanced combination therapy in IBD. The first is in a complicated IBD patient with poorly controlled luminal disease on a biologic therapy with partial response with the intent of adding a second biological therapy to control the luminal disease and have a complete response. However, the other scenario is double indication in an IBD patient with concomitant EIM or IMID where one of the biological therapies is controlling the IBD and we add a second biological therapy to control the EIM or IMID [77]. 

A specific question is vedolizumab. Some years ago, multiple cohort studies suggested that the use of vedolizumab may be associated with the exacerbation or worsening of EIMs. Ramos et al. (Inflammatory Bowel Diseases, 2021) conducted a multicenter retrospective cohort study involving 201 patients with inflammatory bowel disease (IBD) and pre-existing extraintestinal manifestations (EIMs) who were treated with vedolizumab [137]. Worsening of EIM occurred in 34.8% of patients. Peripheral arthritis was significantly more common in the worsening group (84.3% vs. 59.6%, *p* < 0.01) [137]. 

A major limitation on the strength of recommendation is that specific studies in patients with coexisting IBD and IMID/EIMs are missing; however, the recent clinical trials place a special emphasis on the improvement.

## 6. Conclusions

There was a revolution in the treatment of IBDs and associated IMIDs/EIM conditions in the last 2 decades with the advent of the biological era. We are able to offer superior disease control to a significant number of patients. However, concomitant IMIDs/EIM represents in many cases a significant clinical challenge and the optimal management of IBD with associated IMIDs is best managed in a multidisciplinary approach, where there is close cooperation among specialists to align treatment goals and management plans for each underlying condition.

## Figures and Tables

**Table 1:** Organ system-based classification of classic EIMs and associated IMIDs in IBD.

System	EIMs	IMIDs	References
Dermatological	Erythema nodosum, Pyoderma Gangrenosum	Psoriasis, Hidradenitis suppurativa	[9,10]
Rheumatological	Axial Spondylarthritis, Ankylosing spondylitis, peripheral arthritis.	Rheumatoid arthritis, Psoriatic arthropathy	
Ophthalmological	Episcleritis, scleritis, uveitis		
Hepatobiliary	Primary sclerosing cholangitis (PSC)		
Neurological		Multiple Sclerosis	

**Table 2:** Medical therapies for IBD and IMIDs/EIM.

Drug	IBD Indication	IMIDs/EIM	Reference
5-ASA (mesalamine, sulfasalazine)	UC maintenance	Rheumatoid arthritis (sulfasalazine only)	[11]
Azathioprine	UC/CD Maintenance	Rheumatoid arthritis	[10,12,13]
Methotrexate	CD Maintenance	Psoriasis, psoriatic arthritis, Rheumatoid arthritis	[14,15,16,17,18]
Infliximab	UC/CD	Psoriasis, psoriatic arthritis, Rheumatoid arthritis, uveitis, EN, HS, PG, axial SpA Contraindication: MS	[19,20,21,22,23,24,25,26,27]
Adalimumab	UC/CD	Psoriasis, psoriatic arthritis, Rheumatoid arthritis, uveitis, EN, HS, PG, SpA. Contraindication: MS	[22,26,28,29,30,31,32,33]
Certolizumab	CD	Psoriasis, psoriatic arthritis, Rheumatoid arthritis, uveitis, SpA. Contraindication: MS	[29,34,35,36,37]
Golimumab	UC	SpA, psoriatic arthritis, uveitis, RA. Contraindications: MS	[13,37,38,39,40]
Vedolizumab	UC/CD	Possible Erythema nodosum (low mixed evidence)	[41,42]
Ustekinumab	UC/CD	Psoriasis, peripheral SpA, psoriatic arthritis	[43,44,45]
Risankizumab	UC/CD	Psoriasis, psoriatic arthritis	[46,47,48,49]
Guselkumab	UC/CD	Psoriasis, psoriatic arthritis	[29,50,51,52]
Mirikizumab	UC/CD	None	[53,54,55,56]
Tofacitinib	UC	Psoriasis, psoriatic arthritis, Rheumatoid arthritis, SpA	[57,58,59,60,61]
Upadacitinib	UC/CD	Psoriasis, psoriatic arthritis, Rheumatoid arthritis, SpA	[62,63,64,65,66]
Ozanimod	UC	MS	[67,68]
Etrasimod	UC	None	[69]
Natalizumab	CD	MS	[70,71]

Acronyms: 5-ASA: 5-aminosalicyclic acid, UC: ulcerative colitis, CD: Crohn’s disease, EN: erythema nodosum, HS: Hidradenitis suppurative, PG: Pyoderma gangrenosum, MS: Multiple Sclerosis, SpA: Spondyloarthritis, RA: Rheumatoid arthritis.

**Table 3:** Safety profile and common adverse effects of advanced therapy.

Advanced Therapy	Common Adverse Effects	
Anti-TNF (infliximab)	upper respiratory tract infection (23.8%), arthralgia (17.2%), headache (14.8%), sinusitis (13.1%), pharyngitis (11.5%)	[72]
IL12/23 inhibitor (Ustekinumab)	Headache (6.9%), anemia (2.2%), upper respiratory tract infection (1.9%), pyrexia (1.2%), arthralgia (0.9%),	[43]
IL 23p19 inhibitor (Risankizumab)	nasopharyngitis (23.4%), upper respiratory tract infection (15.4%), headache (6.8%), arthralgia (9%), back pain (6%)	[73]
JAK inhibitor (upadacitinib)	nasopharyngitis (5%), CPK elevation (5%), acne (5%), upper respiratory tract infection (3%), arthralgia (2%)	[48]
S1P- modulator (etrasimod)	Anemia (8%), headache (8%), dizziness (5%), pyrexia (5%), arthralgia (4%) nausea (3%), herpes zoster (1%), bradycardia (1%), AV block (<1%).	[69]
Anti-integrin (Vedolizumab)	Nasopharyngitis (28.2%), upper respiratory tract infection (18.6%), headache (18.3%), abdominal pain (12.4%), arthralgia (17.3%), pyrexia (8.9%).	[74]
Azathioprine	Nausea (8%), leukopenia (4.1%), hepatotoxicity (4%), pancreatitis (4%), bone marrow failure (2.2%), infections (1.7%), arthralgia (1.2%), fever (1%), anemia (0.9%), headache (0.3%), cancer (0.2), lymphopenia (0.1%)	[75]
Methotrexate	Nausea/vomiting (17%), weakness (11%), liver toxicity (8%), skin reactions (2%), paresthesia (2%), infections (2%), leukopenia (1%), pneumonitis (1%).	[76]

**Table 4:** Advanced therapies for psoriasis/psoriatic arthritis with IBD.

	Advanced Therapy	Avoided Advanced Therapy
Psoriasis with IBD	Anti-TNF agents (except Etanercept), IL12/23 inhibitor (ustekinumab), IL-23 inhibitor (risankizumab and guselkumab).	Etanercept, Anti-IL-17.
Psoriatic arthritis with IBD	IL-23 inhibitor (risankizumab and guselkumab), IL-12/23 inhibitor (Ustekinumab), Anti-TNF (except etanercept), JAK inhibitors (tofacitinib, Upadacitinib)	Etanercept, Anti-IL-17

**Table 5:** Advanced therapies for rheumatoid arthritis with IBD.

	Advanced Therapy	Avoided Advanced Therapy
Rheumatoid arthritis with IBD	Anti-TNF agent (except etanercept), JAK inhibitor (upadacitinib and tofacitinib)	Etanercept, Anti-CD20 (rituximab), Anti-IL17.

**Table 6:** Advanced therapies for multiple sclerosis with IBD.

	Advanced Therapy	Avoided Advanced Therapy
Multiple sclerosis with IBD	Ozanimod, Natalizumab.	Anti-TNF agents, Anti-CD 20.

**Table 7:** Advanced therapies for hidradenitis suppurative with IBD.

	Advanced Therapy	Avoided Advanced Therapy
Hidradenitis suppurative with IBD	Adalimumab, Infliximab, Ustekinumab	IL-17 inhibitor

**Table 8:** Advanced therapies for SpA with IBD.

	Advanced Therapy	Avoided Advanced Therapy
Axial SpA with IBD	Anti-TNF agents (except etanercept), JAK inhibitor (upadacitinib, tofacitnib)	Etanercept, IL-17 inhibitor.
Peripheral SpA with IBD	Anti-TNF agents (except etanercept), IL12/23 inhibitor (ustekinumab), IL-23 inhibitor (risankizumab, guselkumab), JAK inhibitor (upadacitinib, tofacitnib).	Etanercept, IL-17 inhibitor.

**Table 9:** Advanced therapies for erythema nodosum with IBD.

	Advanced Therapy	Avoided Advanced Therapy
Erythema nodosum with IBD	Anti-TNF agents (adalimumab and infliximab), IL-12/23 inhibitor (Ustekinumab)	Etanercept

**Table 10:** Advanced therapies for pyoderma gangrenosum with IBD.

	Advanced Therapy	Avoided Advanced Therapy
Pyoderma gangrenosum with IBD	Infliximab	Etanercept

**Table 11:** Advanced therapies for episcleitis/scleritis with IBD.

	Advanced Therapy	Avoided Advanced Therapy
Episcleitis/scleritis with IBD	Anti-TNF (specifically Infliximab, Adalimumab)	Etanercept

**Table 12:** Advanced therapies for uveitis with IBD.

	Advanced Therapy	Avoided Advanced Therapy
Uveitis with IBD	Adalimumab	Etanercept

## Data Availability

No new data were created or analyzed in this study.

## Abbreviations

The following abbreviations are used in this manuscript:

IBD	Inflammatory bowel disease
UC	Ulcerative colitis
CD	Crohn’s disease
IMIDs	Immune mediated inflammatory disease
SpA	Spondyloarthritis
AS	Ankylosing spondylitis
MS	Multiple Sclerosis
HS	Hidradenitis suppurative
EN	Erythema nodosum
PG	Pyoderma gangrenosum
RA	Rheumatoid arthritis
PSC	Primary sclerosing cholangitis
5-ASA	5-aminosalicyclic acid
Ps	Psoriasis
PsA	Psoriatic arthritis
EIM	Extraintestinal manifestation
TNF	Tumor necrosis factor
IL	Interleukin
PASI	Psoriasis area and severity index
ACR	American college of rheumatology
DMARD	Disease modifying antirheumatic drug
NSAID	Non steroidal anti-inflammatory drugs
JAK	Janus kinase
PML	Progressive multifocal leukoencephalopathy
JCV	John Cunningham virus
HiSCR	Hidradenitis suppurative clinical response
HLA-B27	Human leukocyte antigen B27
